# RhoA and Rac1 in Liver Cancer Cells: Induction of Overexpression Using Mechanical Stimulation

**DOI:** 10.3390/mi11080729

**Published:** 2020-07-28

**Authors:** Sharda Yadav, Navid Kashaninejad, Nam-Trung Nguyen

**Affiliations:** Queensland Micro- and Nanotechnology Centre, Nathan Campus, Griffith University,170 Kessels Road, Brisbane, QLD 4111, Australia; sharda.yadav@griffithuni.edu.au (S.Y.); n.kashaninejad@griffith.edu.au (N.K.)

**Keywords:** liver cancer, HCC, Rho GTPase, cell-stretching, mechanobiology, Rac1, RhoA

## Abstract

Liver cancer, especially hepatocellular carcinoma (HCC), is an aggressive disease with an extremely high mortality rate. Unfortunately, no promising markers are currently available for the early diagnosis of this disease. Thus, a reliable biomarker reflecting the early behaviour of the tumour will be valuable for diagnosis and treatment. The Ras homologous (Rho) GTPases, which belong to the small guanosine triphosphate (GTP) binding proteins, have been reported to play an important role in mediating liver cancer based on their important function in cytoskeletal reorganisation. These proteins can be either oncogenic or tumour suppressors. They are also associated with the acquirement of malignant features by cancer cells. The overexpression of RhoA and Rac1, members of the Rho GTPases, have been linked with carcinogenesis and the progression of different types of cancer. In the quest of elucidating the role of mechanical stimulation in the mechanobiology of liver cancer cells, this paper evaluates the effect of stretching on the expression levels of RhoA and Rac1 in different types of liver cancers. It is shown that that stretching liver cancer cells significantly increases the expression levels of RhoA and Rac1 in HCC and cholangiocarcinoma cell lines. We hypothesise that this relatively simple and sensitive method could be helpful for screening biological features and provide suitable treatment guidance for liver cancer patients.

## 1. Introduction

Liver cancer is one of the most aggressive malignancies, with an estimated more than 31,000 deaths in 2019. Liver cancer is the fifth most common cause of cancer death for men and the seventh, for women. The overall mortality rate more than doubled from 1980 to 2016 [1,2]. Previously, liver cancer was most prevalent in Asian and African regions. However, it is rapidly becoming more prevalent in Western countries due to an increase in the hepatitis C infection rate and cancers associated with alcohol and non-alcoholic steatohepatitis [3,4,5]. Consequently, liver cancer has emerged as a global health problem.

There are generally two types of liver cancer: primary and secondary. Primary liver cancer originates from the various cells that make up the liver. The most common type of primary liver cancer is hepatocellular carcinoma (HCC), which accounts for 85–90% of primary liver cancers. In more than 80% of cases, HCC occurs because of chronic liver injury and cirrhosis [6,7,8]. Another type of primary liver cancer is cholangiocarcinoma, which develops in the small, tube-like bile ducts in the liver and accounts for approximately 10 to 20% of all liver cancers [9]. Developments in the diagnosis and management of HCC have a substantial impact on patients who are at risk of developing HCC. The secondary type of cancer in the liver is the result of the metastasis of circulating tumour cells (CTCs), originating from the primary tumours of organs of the body other than the liver. The recent approaches in the early detection of CTCs and CTC clusters, which can lead to the early diagnosis of secondary liver cancer, were thoroughly discussed by Rostami et al. [10]. 

Currently, several methods have been put forth for the diagnosis of liver cancers. Serological markers such as total α-fetoprotein (AFP) and lens culinaris agglutinin-reactive AFP (AFP-L3) are available. Additionally, radiology, which uses imaging techniques such as ultrasound, computerised tomography (CT) scans, hepatic angiography and magnetic resonance imaging (MRI), has been routinely utilised to diagnose liver cancers [11]. Nevertheless, liver biopsy is still considered as the “golden standard” technique for the diagnosis of liver cancer. However, this technique is highly invasive and poses significant risks. Recent developments in diagnostic modalities such as imaging technologies such as positron emission tomography and serum markers have improved the rate of early diagnosis. However, HCC is still one of the major cancers with a high mortality rate. Thus, reliable biomarkers reflecting the early behaviour of the tumour will be valuable for the detection and treatment of liver cancer.

Stimulus-responsive biomarkers that control signal transduction pathways can potentially be used to diagnose HCC at its early stage. It is well known that guanosine triphosphate (GTP) is crucial for the protein synthesis and transcription process. In the context of the cellular response to external stimuli, a group of hydrolase enzymes, called GTPases, that convert GTP to guanosine diphosphate (GDP) are responsible for signal transduction. Specifically, the Ras homologous (Rho) GTPases are a subfamily of small G proteins that play a crucial role in cancer metastasis and invasion. They directly affect actin reorganisation and cytoskeletal arrangement, which are essential for cell movement. Recently, these proteins have been considered as a cancer-related biomarker [12,13,14,15]. Rho GTPases regulate cell morphology through the cytoskeleton and participate in the regulation of the polarity of the cancer cells. They also have marked roles in the migration and proliferation of cancer cells [16]. The extensively explored members of this family are RhoA (Ras homolog family member A), Cdc42 (cell division control protein 42 homolog) and Rac1 (Ras-related C3 botulinum toxin substrate 1) [17,18]. RhoA is activated by either mechanical stress or by ROCK (Rho-associated protein kinase) or mammalian diaphanous-related formin. Similarly, mechanical stimuli activate Rac1, which alters the orientation of stress fibres and influences the cells subject to periodic stretching.

Furthermore, Rho proteins play a significant role in carcinogenesis and cancer metastasis [19]. Rho A assists in the generation of stress fibres and focal adhesions (via the focal adhesion points) of cells. Meanwhile, Rac1 plays a vital role in regulating the actin cytoskeleton by activating p21- activated kinases, promoting cell proliferation through the MAPK (mitogen-activated protein kinase) system. These functions of RhoA and Rac1 become crucial for angiogenesis and tumour initiation, invasion and metastasis. Due to this, the potential role of RhoA and Rac1 in tumour development has attracted a great deal of attention from the research community in cancer biology [20,21]. Furthermore, previous reports have explained the increased concentrations of the RhoA protein in breast, lung and colon cancer. This suggests that RhoA plays an essential role in the invasion of neoplasms [22]. Besides, the increased expression of Rac1 has been observed in many types of cancer, such as gastric, oesophageal, gallbladder, lung, hepatocellular, breast and ovarian cancer [14]. Recently, studies have also focused explicitly on the role of Rho GTPases activating proteins with implications in HCC [23]. The high interaction of Rho GTPases with the extracellular matrix (ECM) also signifies their role in producing the components of the ECM through mechanotransduction [24]. Rho GTPases such as RhoA and Rac1 are activated via the Hippo pathway and inhibit Lats1/2 kinase activity. The Hippo pathway translates the signal from the extracellular matrix to the intracellular matrix when the integrins are activated.

Innovative technology platforms have shown tremendous promise in cancer management [25,26,27]. Recently, our group reported that stretching could induce the overexpression of GTPases in breast cancer cells [28]. We observed high indirect involvements of Rho GTPases with the ECM of breast cancer cells. The ECM is highly involved in cancer due to penetrating cells. Thus, these cells continuously express the Rho GTPases to counteract the strain induced by the ECM. Probing these forces in breast cancer by simulating the ECM force has supported the hypothesis that more GTPases are released by applying strain to cells [28]. Apart from the detection of the potential metastatic transformation of cells, Rho proteins and the pathways they follow can be utilized for therapeutic development for cancer. The screening of the levels of these GTPases and their blockade provide import insights into cancer progression. Cell stretching platforms accelerate the process of these kinases being released in cancer and hence accelerate the testing of the therapeutics. These stretching platforms are also facile methods for mimicking the stress on the cells for fundamental cell biological studies in cancer.

In this study, we investigate whether the mechanical stretching of liver cancer cells can lead to the overexpression of Rho A and Rac1. To this end, the liver cancer cell lines were seeded on a membrane placed in a magnetically actuated cell stretching device and the protein expression level was subsequently quantified by using enzyme-linked immunosorbent assay (ELISA). Herein, we used four different types of liver cancer cells, namely, the PLC/PRF/5, HepG2, SNU 245 and Hep 3B cell lines. This platform can amplify the protein signalling in liver cancer cells, realising a simple and sensitive technique for detecting liver cancer protein biomarkers.

## 2. Materials and Methods

### 2.1. An Electromagnetic Actuation Device for Cell Stretching

The details of the electromagnetic actuated device used for cell stretching has been explained in our previous publications [29,30]. Briefly, the device includes a mounting stage with electromagnets for cyclic strain, a holding clip, and a polydimethylsiloxane (PDMS) membrane with embedded permanent magnets. This platform has been specifically designed for mechanobiology research. The cells seeded on the device can experience both static strain and cyclic stretching conditions. The base of the device is made from a PDMS membrane that contains two permanent magnets embedded on opposite sides of the device. The permanent magnets are 15 mm in diameter and 2 mm thick and are made up of NdFeB alloy. To induce the required deformation of the PDMS membrane, the north poles of both magnets are 8 mm apart in front of each other to produce the corresponding repulsive force.

Another part of the device is a deformable PDMS membrane with a 200 µm thickness, which is bonded to the main PDMS frame using oxygen plasma treatment. This PDMS device is mounted on an electromagnetic stage using a custom-made holding clip to induce cyclic strain conditions. The clip also helps to compensate the repulsive forces of the magnets and help the device to stay in the intended orientation. The electromagnet (JL Magnet, JL Magnet Co., Ltd., Seoul, Korea) is controlled by a programmable DC power supply (MK Power, M-K Power Products Corporation., Mississauga, ON, Canada), which serves as the actuator in the system. In each experiment, the cells seeded on the deformable membrane were periodically stretched. Once the stretching is started, the electromagnets and permanent magnets produce the magnetic forces and deform the wall of the PDMS device. The force is shifted to the thin deformable membrane, which induces the defined strain on the cultured cells.

The full investigation of the effects of different parameters on the cell stretching platform was initially performed during the design of the system by Kamble et al. [30]. The team optimized parameters such as the force applied, strength of strain or strain rate and different types of stretch conditions to simulate the natural environment. The work demonstrated that an input voltage of 27 V for both actuators provided an average homogeneous cyclic strain of 1.38 ± 0.021% over the central region of the membrane. The force optimizations were carried out from 0.5 to 1 N with increments of 0.5 mN. The results were optimised by simulations and verified by experimental data. Additionally, the team studied the effects of cyclic and static strain. Cyclic stretching was more native to tissue-like strain than static strain. In this work, we adapted those optimised parameters for the experimental design.

### 2.2. Seeding and Culturing Liver Cancer Cells on the Deformable Membrane of the Electromagnetic Device

All the liver cancer cell lines (PLC/PRF/5, HepG2, Hep3B and SNU-245) were acquired from the American Type of Culture Collection (ATCC). All these cancer cells are hepatocellular cancer cells except for SNU-245, which is a cholangiocarcinoma cell line. Cholangiocarcinoma begins in the bile ducts, which are branched tubes that connect the liver and gallbladder to the small intestine. Because it occurs in the parts of the bile ducts within the liver, cholangiocarcinoma can also be classified as a type of liver cancer. PLC/PRF/5 (Alexander) cells are a human liver cancer cell line that synthesises the hepatitis B virus antigen (HBsAg). These cells were isolated from a primary liver carcinoma. HepG2 had been derived from the liver tissue of a patient with a well-differentiated hepatocellular carcinoma. This cell line responds to stimulation with human growth hormone. Hep3B had been derived from the liver tissue of a patient with an integrated hepatitis B virus genome. SNU-245 had been isolated from extrahepatic bile duct cancers [31].

To support the growth of the cells, the cancer cells were maintained in DMEM/F12 (Dulbecco’s Modified Eagle Medium/Nutrient Mixture F-12) medium (Gibco, Thermo Fisher Scientific, Waltham, MA, USA). The medium was also supplemented with 10% fetal bovine serum (FBS) and 1% penicillin/streptomycin to prevent infection. All the cells were grown in a culture flasks (T75) and kept in an incubator at 37 °C with 5% CO_2_. The device was sterilised with 80% ethanol and washed (3×) with sterile 1× Hank’s balanced salt solution (HBSS), followed by 30 min of ultraviolet (UV) irradiation. Before seeding, the PDMS membrane (8.78 mm) was treated with 400 µL of DMEM-F12 medium and incubated for one hour to further enhance its biocompatibility. Once the cells reached 80% confluence, they were harvested from the flasks and counted with a haemocytometer. In total, we seeded 75,000 cells onto the PDMS membrane. The cells were placed inside the incubator for 24 h to optimise their adhesion and growth on the PDMS membrane. Subsequently, the cultured cells were washed (3×) with HBSS and topped up with 400 µL of medium. Next, the mechanical strain was applied as explained in following section.

### 2.3. Applying Cyclic Stretching on the Cultured Cells

The PDMS membrane was mounted on the electromagnetic platform and placed in a 5% CO_2_ environment at 37 °C, maintaining the physiological temperature. As optimised in our previous studies, the cells were subjected to 1.4% strain at 0.01 Hz and 50% duty cyclic stretching. The strain applied to the deformable membrane had been characterised, both experimentally and numerically [30]. The voltage range of 1 to 30 V was applied to the membrane, and the deformation of the membrane was recorded with a digital camera (EO Edmund). We applied the related strain over two different intervals, i.e., 2 and 4 h. Next, the cells were lysed to investigate the amounts of released biomarkers. The procedure used for cell lysis will be explained in the next section.

### 2.4. Cell Lysis Procedure

**Figure A1:**
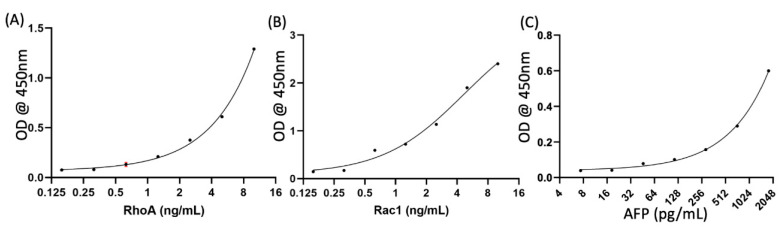
(**A**) The standard curve for the quantification of RhoA (R. sq. 0.996). (**B**) The standard curve for quantification of Rac1 (R. sq. 0.990). (**C**) The standard curve for quantification of AFP (R. sq. 0.998).

After the completion of the stretching cycles, the cells were harvested, first, by washing (3×) followed by trypsin addition to dislodge the cells from the membrane. The cells were subsequently centrifuged, pelleted and counted before chemical lysis. To lyse the cells, we used 1% NP-40, 50 mM Tris-HCl, 20 mM ethylenediaminetetraacetic acid (EDTA), pH 7.5. The cells and the lysis buffer were incubated on ice for 30 min. Then, the lysate was kept for centrifugation for 5 min at 5000 RPM for separating the cell debris by pelleting. The supernatant with protein markers was removed in a separate aliquot for further quantification by ELISA. To subsequently use the lysed cells, we collected and stored the supernatant at −20 °C. The chemical disruption of the cells using NP-40 we found to be the best method for releasing RhoA and Rac1 from the cells in comparison to heat shock and sonication-based lysis methods (Figure A1).

### 2.5. Quantification and Detection of RhoA and Rac1 Biomarkers Using ELISA Technique

The levels of RhoA and Rac1 were measured using the RHOA ELISA Kit and RAC1 ELISA Kit, respectively, purchased from MyBioSource, Inc. San Diego, CA, USA. We performed the technique based on the standard protocol. Briefly, 100 µL of the solution containing the lysed cells was added to pre-coated 96-well plates, i.e., 100 µL/well. The plates were placed inside an incubator for 1 h. Then, the detection reagent A (100 µL) was added to the solution and the plates were incubated again for another 1 h. After subsequent washing (3×), the detection reagent B (100 µL) was added to the substrate and kept for incubation at 37 °C for 30 min. The plates were washed (3×), 90 µL of horseradish peroxidase (HRP) substrate solution was added, and the plates were further incubated for 20 min. The enzyme–substrate reaction was terminated by adding 50 µL of stop solution. A microplate reader (SpectraMax) at a wavelength of 450 nm was used to detect the colour changes. Finally, the optical density (OD) of the samples was compared to that of the standard curve. Using such OD differences, we quantified the concentrations of RhoA and Rac1 in the samples. The marker protein human alpha-fetoprotein (AFP), not involved in cytoskeletal functions, was chosen as a control. The level of this protein was determined with an ELISA Kit purchased from Sigma-Aldrich, Australia, following the manufacturer’s instructions.

### 2.6. Statistical Analysis

We used the Graphpad Prism 8 software (GraphPad Software, San Diego, CA, USA) to perform the statistical analysis of all the experiments. All the reported data are the averages of three repetitions. The quantities of RhoA, Rac1 and AFP were normalised against the cell counts. We used Student’s *t*-test to calculate the related *p* values. The obtained results were reported as statistically significant when the calculated *p* values were less than 5%.

### 2.7. Immunofluorosence Staining

We used standard immunofluorosence staining to observe the actin filaments and nuclei of the cells seeded on the PDMS membrane before and after stretching. To this end, the cells were first fixed with 4% paraformaldehyde (PFA) for 30 min, followed by washing with HBSS (3×). Next, the cells were stained with ActinGreen^TM^ 488 (Thermo Fisher Scientific) and NucBlue^TM^ ReadyProbe^TM^ reagents (Thermo Fisher Scientific) and kept for incubation at room temperature for 30 min (based on the manufacturer’s recommendations). Finally, the stained cells were washed with HBSS (3×).

### 2.8. Fluorescence Microscopy

We first cut the PDMS membrane containing the stained cells (the immunostaining procedure is explained in the previous section) and placed it directly onto a microscope slide. A fluorescent microscope (Nikon Eclipse Ti2) was used to capture the images of the actin fibres and nuclei of the cells. We used Image J 1.47v (National Institutes of Health, Bethesda, MD, USA) for subsequent image processing.

## 3. Results and Discussion

**Figure 1:**
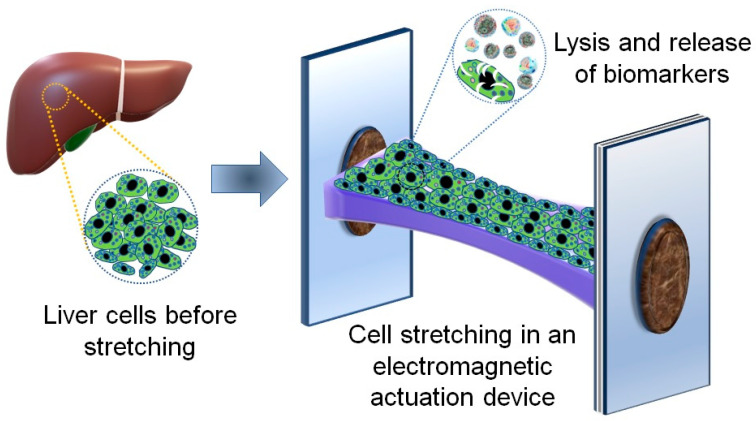
Graphic illustration of the procedure used for the quantification of protein biomarkers (RhoA, Rac1 and ALP) in liver cancer cells. The liver cancer cells were seeded once the deformable membrane was bonded on a magnetically actuated polydimethylsiloxane (PDMS) support. The PDMS parts device was then inserted on the cell stretching platform, followed by the application of the cyclic mechanical strain. The stretched cells were lysed chemically, and the supernatant was collected for the quantification of the released protein biomarkers. The levels of the markers released before and after stretching were quantified by using ELISA.

Three HCC cell lines (PLC/PRF/5, HepG2 and Hep3B) and one cholangiocarcinoma cell line (SNU-245) were subjected to stretching on the electromagnetic platform and cellular lysis as per the workflow depicted in Figure 1. Briefly, the confluent culture of cells was seeded on the thin deformable PDMS membrane. The membrane was incubated for 24 h under standard cell culture conditions—i.e., 37 °C, 5% CO_2_ and 95% humidity—to maintain the optimum environment. Next, we placed the membrane into the periodic stretching platform so that the cells could be subjected to mechanical stimulations. Post stretching, the cells were washed (3×), trypsinised and counted. Although the initial number of the seeded cells was 75,000 cells per assay, only 10,000 cells were recovered following the prolonged stretching procedure. This finding is consistent with our previous work where we observed a significant increase in the number of dead cells after stretching. This can be explained by the rigidity of the cell membranes [29]. Since cancer cells have stiffer cell membranes than healthy cells, they are less tolerant to mechanical stretching. Similar to our observation, cancer cell death proceeded via the apoptotic mechanism during compressive stress, while healthy cells tolerated the same order of compressive stress for up to 4 h [32].

### 3.1. Qualitative Analysis of Effect of Stretching on Liver Cancer Cells

**Figure 2:**
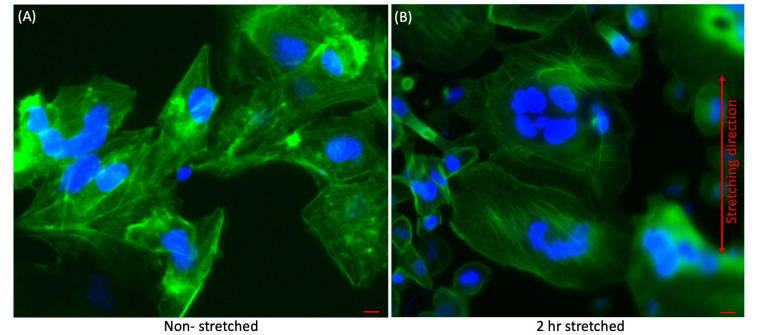
Representative fluorescence images of (**A**) Non-stretched (**B**) 2 h stretched HepG2 cells presenting the morphology and distribution of cells after stretching for 2 h compared with non-stretched cells. ActinGreen (green) labels cellular actin, and Nucblue (blue) stains nuclei (×20). As a result of the mechanical stimulations, the actin stress fibres of the stretched cells gradually reconstructed. Accordingly, the cytoskeleton architecture of the stretched cells was reorganized over time. Both scale bars in the figure are 50 µm.

Figure 2A,B show the representative fluorescence image of HepG2 with the cell morphology and distribution before and after stretching for 2 hr. As a result of the mechanical stimulations, the actin stress fibres of the stretched cells gradually reconstructed. Accordingly, the cytoskeleton architecture of the stretched cells was reorganized over time.

All the viable cells were collected after the stretching to release the protein biomarkers from the cells. The viability of the cells was recorded as per the procedure described by us in Sharda et al. [29]. Briefly, flow cytometric analysis was performed to differentiate live and dead cells using acridine orange and propidium iodide (PI) dyes. The propidium dye only enters dead cells, enabling their differentiation. The percentage of PI-positive cells was 69.90% in the four-hour-stretched cells compared to only 14.20% in the non-stretched samples. Since RhoA and Rac1 are intracellular markers, membrane disruption is required to release these markers into the sample for further quantification by ELISA. As explained in Section 2.4, we used a detergent-based lysis technique to break the hydrophobic–hydrophilic interactions of the cells, achieved by the use of the mild surfactant NP-40. This surfactant is commonly used to investigate cytoplasmic or membrane-bound proteins.

### 3.2. Quantitative Analysis of Effect of Stretching on Liver Cancer Cells

To quantitatively study the effect of cell stretching, we measured the concentrations of the released RhoA and Rac1 biomarkers by ELISA. To this end, the samples were added to the respective microplate wells with a biotin-conjugated antibody specific to RhoA and Rac1. Afterwards, avidin conjugated to HRP was added to each microplate well and kept for incubation. Next, the TMB substrate (3,3′,5,5′-tetramethylbenzidine) was added and kept in a dark place. After the addition of the substrate, only those wells that contained RhoA and Rac1, biotin-conjugated antibody and HRP-conjugated avidin changed the colour. Subsequently, a mild sulphuric acid solution was used for the termination of the enzyme–substrate reaction. Finally, a microplate reader was used to detect the concentrations of RhoA and Rac1, as explained in Section 2.5.

#### 3.2.1. Difference between the Concentrations of RhoA and Rac1 before and after Applying Mechanical Strain

The difference in the expression levels of Rho proteins before and after stretching in breast cancer cells had already been observed, and the results were reported in our previous publication [28]. Therefore, to determine whether the expression of the RhoA protein in liver cancer cells was also affected by cyclic stretching, we measured the relative expression levels of RhoA before and after applying the strain on the HCC cell line. We stretched the PLC/PRF/5 cells for 2 hr, followed by the chemical lysis of cells, the release of the RhoA markers, and their quantification using ELISA. It was observed that the level of RhoA increased from 0.05 to 0.15 pg/mL following 2 h of cyclic stretching. Since high levels of expression of the RhoA protein in breast, colon and lung cancers have been also reported in the literature, it can be concluded that RhoA has a crucial role in the invasive process of neoplasms [24]. It is important to remark that there was a clearly visible difference in the expression level of RhoA after applying mechanical strain to the cells.

Subsequently, the Rac1 expression levels of the PLC/PRF/5 cells were quantified. For this specific cell line, the level of Rac1 increased from 0.015 to 0.03 pg/mL after 2 h of periodic mechanical strain. The expression level of both markers was increased after 2 h of stretching; however, the difference was not clearly reflected within this time frame.

#### 3.2.2. Time-Dependent Expression of RhoA

**Figure 3:**
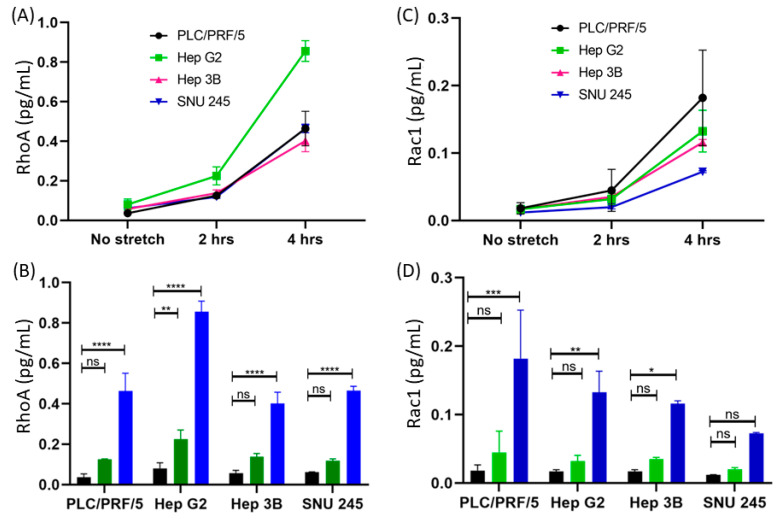
Time course of expression of RhoA and Rac1 before and after applying the strain in various types of liver cancer cells. We measured the released biomarkers after multiple durations of mechanical stretching, i.e., 0 (control), 2 and 4 h. To quantify the levels of expression of RhoA and Rac1 in response to cyclic strain, the ELISA detection method was used. (**A**) Quantitative measurement of RhoA activity in multiple cell lines pre and post stretching for 2 and 4 h; (**B**) quantification of RhoA before and after the stretching for 2 and 4 h of PLC/PRF/5, HepG2, Hep3B and SNU-245 liver cancer samples; (**C**) measurement of Rac1 quantity in multiple cell lines pre and post stretching for 2 and 4 h; (**D**) quantification of Rac1 pre and post the stretching for 2 and 4 h of PLC/PRF/5, HepG2, Hep3B and SNU-245 liver cancer samples. The *p* values were obtained using Student’s *t*-test. (*): *p* ≤ 0.05; (**): *p* ≤ 0.005, (***): *p* ≤ 0.0005 and (****): *p* ≤ 0.0001. The error bars show the standard deviations of the experiments (*n* = 3).

We also investigated the time-dependent change in the protein expression levels in the PLC/PRF/5 cells following the continuous periodic strain. To this end, we continued to stretch the cells for 4 h to observe the time-dependent activation of the RhoA biomarker. Figure 3A shows the same trend of protein expression in four different types of liver cancer cells. Initially, there is a rise after the first two hours of stretching, and interestingly, there is a severe increase after prolonged stretching.

#### 3.2.3. Detection of RhoA in Multiple Cell Lines

Along with PLC/PRF/5 cells, we extended our assay with two more HCC cell lines (HepG2 and Hep3B) and one cholangiocarcinoma cell line (SNU-245). HCC is the most common and aggressive type of cancer worldwide and is the leading primary malignancy of the liver.

All the different cells were cultured and stretched following the same procedure. As noticed earlier, the RhoA expression level was markedly higher in all the liver cancer cells after longer stretching, i.e., a four-hour stretch time (Figure 3B). In the PLC/PRF/5 cells, it was noted the RhoA protein level prior to stretching was 0.015 pg/mL; however, after stretching, it increased up to 0.44 pg/mL. In HepG2 cells, the levels were 0.08 pg/mL before and 0.81 pg/mL after stretching. Meanwhile, in Hep3B cells the level of RhoA increased from 0.05 pg/mL before stretching to 0.40 pg/mL post stretching. Similarly, in the cholangiocarcinoma cell line SNU-245, the RhoA level was 0.051 pg/mL prior to stretching, which changed to 0.42 pg/mL after stretching. As we can see in the figure, there is a dramatic increase in the HepG2 cell line after stretching compared to in other cells. This significant increase in the expression level of the RhoA protein marker is attributed to the activation of the Rho GTPase pathway in cancer.

#### 3.2.4. Time-Dependent Expression of Rac1

We noted the variation in the Rac1 protein levels between before stretching and after 2 h of stretching in PLC/PRF/5 cells. Next, we stretched four different (PLC/PRF/5, HepG2, Hep3B and SNU-245) types of liver cancer cells for 4 h to observe the time-dependent activation of Rac1. As illustrated in Figure 3C, the quantity of Rac1 was amplified in the initial 2 h of stretching, and there was a remarkable increase after prolonged stretching. Interestingly, both RhoA and Rac1 showed a similar trend.

#### 3.2.5. Detection of Rac1 in Multiple Cell Lines

As explained in Section 3.2.3, we extended our assay with two more HCC cell lines (HepG2 and Hep3B) and one cholangiocarcinoma cell line (SNU-245) for Rac1 detection. We determined that the level of Rac1 prior to stretching was 0.01 pg/mL and that post stretching was 0.18 pg/mL in PLC/PRF/5 cells. Similarly, in HepG2 cells, the level was 0.01 pg/mL prior to stretching and 0.13 pg/mL post stretching. Additionally, in Hep3B, the Rac1 level was 0.01 pg/mL prior to stretching and 0.12 pg/mL post stretching. Similarly, the cholangiocarcinoma cells SNU-245 had 0.01 pg/mL of Rac1 before and 0.08 pg/mL post stretching. It is important to note that we could differentiate between the expression levels before and after stretching in cancer cells. It is clearly visible that the expression level before stretching was very low, which was too weak to be detected (Figure 3D). However, there was a drastic increase in the expression level after applying mechanical strain. Interestingly, the RhoA and Rac1 markers show similar trends by levels of expression. RhoA and Rac1 are important members of the Rho GTPase family and are highly involved in maintaining cellular geometrical homeostasis. They both act on actin fibres by forming either focal adhesion points or lamellipodia. The morphological changes in actin fibres were verified by fluorescence microscopy (Figure 2).

#### 3.2.6. Time-Dependent Activation of AFP

**Figure A2:**
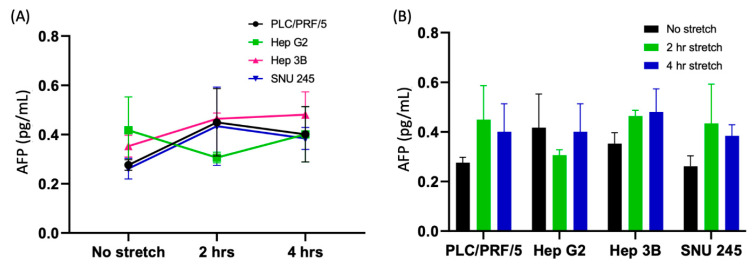
Time course of activation of AFP before and after stretching in four different types of liver cancer cells. We measured the released protein level at different durations of mechanical stretching, i.e., 0 h (control), 2 h and 4 hr. To quantify the level of the activity of AFP in response to stretching, ELISA detection method was used. (**A**) The quantitation of AFP activity in different cell lines before and after stretching for 2 h and 4 hr; (**B**) ELISA detection of protein markers before and after stretching for 2 h and 4 h of PLC/PRF/5, Hep G2, Hep 3B and SNU 245 liver cancer samples.

We further investigated our assay by measuring the protein expression of AFP before and after stretching as a control experiment. The marker protein AFP is not involved in cytoskeletal functions, so it was chosen as a control. As shown in Figure A2A, there was not any significant change in AFP from before to after the stretching of the liver cancer cells.

We noted that the level of AFP prior to stretching was 0.28 pg/mL and that post stretching was 0.38 pg/mL in PLC/PRF/5 cells. Similarly, in the HepG2 cells, the pre-stretching level was 0.38 pg/mL, whereas the post stretching level was 0.42 pg/mL. In the case of Hep3B, the level was 0.38 pg/mL prior to and 0.41 pg/mL post stretching. Similarly, in cholangiocarcinoma cells SNU-245, the level before stretching was 0.23 pg/mL and that after stretching was 0.31 pg/mL. The *p* values acquired using Student’s *t*-test comparing the control and the various durations of mechanical stretching of the four multiple types of liver cancer cells show that the difference is not statistically significant (Table A1).

## 4. Conclusions

In conclusion, we observed the overexpression of Rho protein markers (RhoA and Rac1) in different liver cancer cells when they were stretched for a prolonged period. The assay was also further challenged by measuring the protein expression of AFP, which is not involved in cytoskeletal functions, as a control experiment. This present result correlates well with the earlier studies in that stretching the malignant cells enhances the expression of these markers. The study also suggests that these markers are not limited to only breast cancer (as we showed in our previous publication) but can also be used as a diagnostic biomarker for liver cancer cells. Consistently, the overexpression of the RhoA protein marker has been reported in various cancers, including HCC and colon, testicular, breast, head and neck, lung and bladder cancers [22]. At the same time, elevated levels of Rac1 have been detected in multiple types of malignancies (e.g., gastric cancer, lung cancer, oesophageal cancer, breast cancer, hepatocellular cancer, gallbladder cancer, and ovarian cancer) [21]. In addition to having potential therapeutic value, these protein expression levels are good biomarkers for monitoring the stage of tumour progression. The outcomes warrant future research to determine if the inhibition of Rho GTPases would aid in the spread of cancer or the conversion of hyperplasia to metastasis. A fascial way of performing such studies could be the addition of GTPase-inhibitory drugs in our current platform and the recording of the changes in cellular proliferation. Our stretching strategy could be utilized for screening biological features and providing suitable treatment guidance for cancer patients. This technique could be used to intensify protein signalling and enhance the assay’s sensitivity. Above all, the proposed platform can be considered as an alternative sensitive, simple and low-cost technique for detecting liver cancer biomarkers. We believe that this study offers the advantage of amplifying signals when the signal was tough to quantify.

## Appendix A

### Appendix A.1. Different Cell Lysis Methods

A variety of disruption methods are available for cell lysis, and a few of these methods were tested. Freeze-thaw method is one of the traditional methods for cell lysis which involves freezing a cell suspension in dry ice then thawing the material to room temperature or 37 °C. However, in our experiment heating within a sample led to protein denaturation and aggregation. Another potential option for cell lysis was sonication technique. A sonicator uses pulsed, high-frequency sound waves to agitate the cells. However, this technique also produced cell debris and aggregation, therefore, was not pursued. The mild surfactant NP-40 has an ability to disrupt the cells’ hydrophobic-hydrophilic interactions. It has been a popular buffer for studying proteins that are cytoplasmic or membrane bound. Chemical disruption of cells using NP-40 we found was the best method for releasing RhoA and Rac1 from the cell after testing different lysis techniques.

### Appendix A.3. Quantification of Control Marker AFP

**Table A1:** *p* Values obtained using Student’s *t*-test comparing the control and different durations of mechanical stretching of four different types of liver cancer cells.

S.N.	Comparison	*p* Value	Summary
**1**	**PLC/PRF/5** No stretch vs. 2 h stretch No stretch vs. 4 h stretch	0.5139 0.8373	ns ns
**2**	**Hep G2** No stretch vs. 2 h stretch No stretch vs. 4 h stretch	0.9045 >0.9999	ns ns
**3**	**Hep 3B** No stretch vs. 2 h stretch No stretch vs. 4 h stretch	0.9033 0.8183	ns ns
**4**	**SNU 245** No stretch vs. 2 h stretch No stretch vs. 4 h stretch	0.5188 0.8486	ns ns
